# Role of Neurotransmitters (Biomediators) in Plant Responses to Stress

**DOI:** 10.3390/plants13223134

**Published:** 2024-11-07

**Authors:** Zahra Dehghanian, Mohammad Ahmadabadi, Behnam Asgari Lajayer, Nazila Bagheri, Masoud Chamani, Vahideh Gougerdchi, Mohsen Hamedpour-Darabi, Weixi Shu, G. W. Price, Bernard Dell

**Affiliations:** 1 Department of Biotechnology, Faculty of Agriculture, Azarbaijan Shahid Madani University, Tabriz 53751-71379, Iran; 2 Faculty of Agriculture, Dalhousie University, Truro, NS B2N 5E3, Canada; 3 Department of Plant Protection, Faculty of Agriculture and Natural Resources, University of Mohaghegh Ardabili, Ardabil 56199-11367, Iran; 4 Department of Plant Breeding and Biotechnology, Faculty of Agriculture, University of Tabriz, Tabriz 51666-16471, Iran; 5 Department of Horticultural Science, Faculty of Agriculture, Shiraz University, Shiraz 71946-84471, Iran; 6 Centre for Crop and Food Innovation, Murdoch University, Murdoch 6150, Australia

**Keywords:** abiotic stress, biosynthesis, growth regulation, neurotransmitters, plant signaling

## Abstract

Plants possess a complex signaling system that enables them to sense and adapt to various environmental stressors, including abiotic factors like extreme temperatures, drought, salinity, and toxic heavy metals. While the roles of hormones and signaling molecules in plant stress responses are well established, the involvement of neurotransmitters—traditionally linked to animal nervous systems—in plant stress physiology is a relatively underexplored area. Recent findings indicate that neurotransmitters such as gamma-aminobutyric acid, glutamate, serotonin, and dopamine play crucial roles in several physiological processes within plants. They regulate ion channels, adjust stomatal movements, modulate the production of reactive oxygen species, and influence gene expression. Evidence suggests that these neurotransmitters enhance antioxidant defense mechanisms and regulate stress-responsive pathways vital for plant stress tolerance. Additionally, under stressful conditions, neurotransmitters have been shown to impact plant growth, development, and reproductive activities. This review aims to illuminate the emerging understanding of neurotransmitters as key biomediators in plant responses to abiotic stress.

## 1. Introduction

Plants contain neurotransmitters (NTs) which are essential for physiological functions and cellular communication. These signaling substances include acetylcholine, catecholamines, gamma-aminobutyric acid (GABA), indoleamines, glutamate, and serotonin [1]. Recent studies have revealed that NTs have significant physiological roles in plants (Table 1), including regulation of ion permeability, induction of embryogenesis, enhancement of fruit ripening, delaying senescence, induction of roots and shoots, and protection of germ tissue [2]. In addition, NTs participate in intracellular communications and provide enhanced stress tolerance [1].

The synthesis and roles of NTs in plants and animals have much in common. For instance, the mechanisms for the production of catecholamines, like dopamine, are comparable [43]. In plants, glutamate, a well-known neurotransmitter in animals that also plays important roles in plant growth, development, and response to environmental stresses [44,45], is decarboxylated to produce dopamine in the cytoplasm [1]. The molecular structure of dopamine includes nitrogen and has been studied intensively for its intricate connections in plant systems [46]. As an example, dopamine contents in ripe bananas range from 80 to 560 mg/100 g in the peel, and from 2.5 to 10 mg/100 g in the pulp [47]. Dopamine is also well known for its medicinal benefits in humans [48]. In addition, biogenic amines, including histamine glutamate, an essential amino acid in plant systems that is important for nutrition, metabolism and signaling, are rapidly stimulated in response to stress conditions [1]. Melatonin and serotonin are endogenous chemicals that have evolved to have protective properties [49]. These NTs have been identified in a wide range of plant species [48].

This review aims to clarify the evolving role of neurotransmitters as key biomediators in plant responses to abiotic stressors, highlighting their biochemical pathways and signaling mechanisms in enhancing resilience. By examining recent findings, it advances understanding of plant stress physiology and explores strategies for improving crop tolerance amid global climate change.

## 2. Plant Reaction to Abiotic Stress

**Figure 1:**
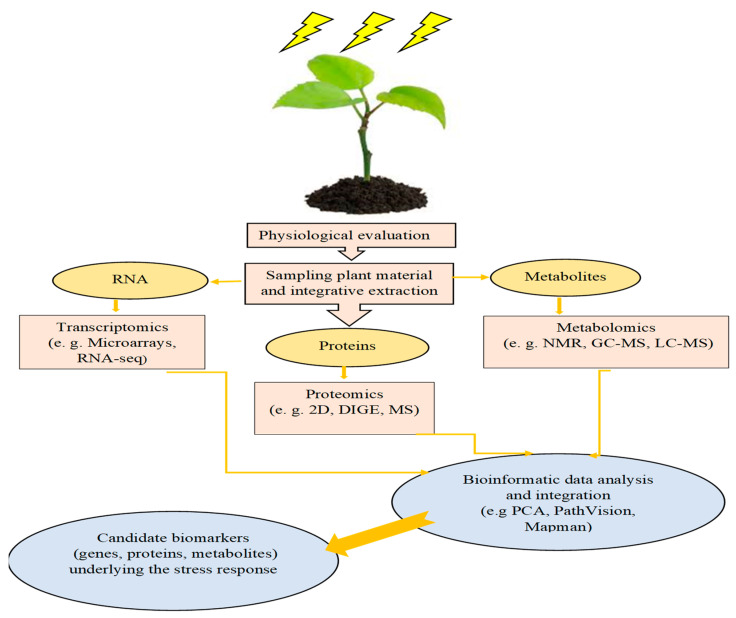
The tools used to explore plant response to abiotic stress (Adapted from Duque et al. [66]).

Since NTs are well known for their role in plant stress tolerance, we briefly introduce plant stress physiology in this section (Figure 1). Crops exposed to adverse environmental conditions produce more reactive oxygen species (ROS), including hydrogen peroxide (H_2_O_2_), hydroxyl radicals (OH^•^), superoxide (O_2_^•−^), and singlet oxygen (1O_2_). ROS detoxification is critical to safeguard plant cells from the destructive effects of abiotic stresses [50,51]. Variations in the specificity of antioxidant enzyme substrates and biochemical pathways, along with distinct gene expression reactions, result in an adaptable and versatile antioxidant response capable of regulating optimal ROS concentrations [52]. Enzymatic and non-enzymatic antioxidants are involved in the ROS scavenging processes [53]. Ascorbate peroxidase (APX) and superoxide dismutase (SOD), occurring as ubiquitous enzymes in the plant cytosol as well as in organelles such as plastids and mitochondria [54,55], are well known for their key role in ROS scavenging and detoxification [56,57,58]. Non-enzymatic antioxidants such as ascorbic acid (AsA) and glutathione (GSH) are also central to crop defense against oxidative stress, acting as antioxidant buffer solutions [59]. Other compounds that may be involved include flavonoids, carotenoids, phenols, alkaloids, and tocopherol [54]. In addition, plants use several other physiological and biochemical mechanisms such as modulation of root architecture [60], regulation of stomatal function [61,62], bio-accumulation of osmoprotectants [63], and early maturation and flowering [64] to withstand stress. NTs have also been documented to regulate these processes. For instance, dopamine has been reported to contribute to stomatal adjustment, resulting in a reduction in the drought stress impact on plant growth [65].

## 3. Dopamine

### 3.1. Synthesis and Degradation

**Figure 2:**
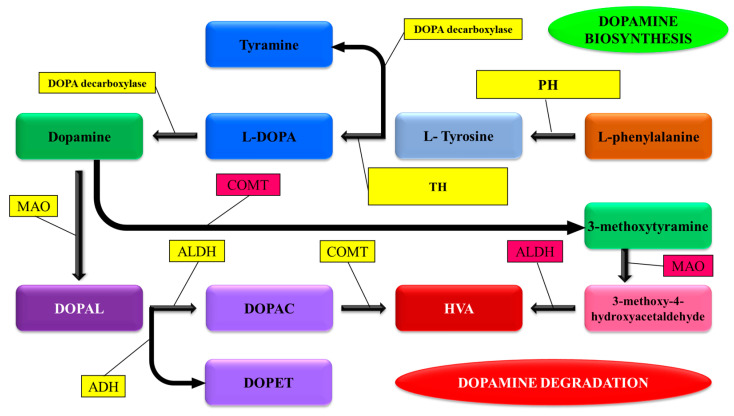
The biosynthesis and degradation pathways of dopamine in plants. MAO: Monoamine oxidase; COMT: Catechol-*O*-methyl transferase; ALDH: Aldehyde dehydrogenase; DOPAC: 3,4-Dihydroxyphenylacetic acid; DOPA: Dihydroxyphenylalanine; DOPET: 3,4-dihydroxyphenylethanol; DOPAL: 3,4-Dihydroxyphenylacetaldehyde; TH: Tyrosinase or Tyrosine hydroxylase; HVA: Homovanillic acid; PH: Phenylalanine hydroxylase (Adapted from Zahoor et al. [69]).

Plants contain two main catecholamine biosynthetic pathways (Figure 2), which are comparable to those found in mammalian species, with tyrosine as the precursor [67]. The first involves tyrosine decarboxylation to build tyramine, followed by hydroxylation by monophenol hydroxylase to produce dopamine (DOPA). In the second pathway, levodopa is first produced by tyrosine hydroxylation, which is subsequently converted to dopamine by DOPA decarboxylase [68]. However, the dopamine biosynthesis pathways differ among plants. Dopamine can be catabolized through methylation or oxidation, resulting in the production of various derivatives. Catechol-O-methyl transferase (COMT) converts melatonin to 3-methoxytyramine, which is further converted to 3-methoxy-4-hydroxyacetaldehyde and homovanillic acid. In addition, melatonin is hydroxylated by monoamine oxidase to produce 3,4-dihydroxyphenylethanol (DOPAL), which is ultimately converted to homovanillic acid by aldehyde dehydrogenase and catechol-*O*-methyl transferase [69].

Methylation of catecholamines can lead to the synthesis of derivative compounds. Extensive studies of the catecholamine metabolism in Dona Ana cactus (*Coryphantha macromeris)* reveal production and accumulation of various methylated catecholamine derivatives. Of these phenethylamines, normacromerine (N-methyl-3,4-dimethoxy-beta-hydroxyphenethylamine) is by far the most abundant [4]. Studies of plant tissue cultures grown in the presence of labelled tyramine and dopamine showed that catecholamines are catabolized also via oxidation and oxidative polymerisation [13]. The plant amine oxidases [14] act indiscriminately on monoamines, oxidising them to the corresponding aldehydes, and thus participate in amine degradation. One of the more important chemical changes is dopamine oxidation by lipoxygenase leading to melanins [15].

### 3.2. Content in Plant Tissues

DOPA concentrations differ greatly among plant species (Table 2), ranging from nanograms to micrograms per gram. For instance, higher DOPA concentrations occur in the pulp of yellow (*Musa acuminata*) and red (*Musa acuminata × M. balbisiana*) bananas, as well as in the fruit peel of Cavendish banana [46]. Lower concentrations of DOPA have been reported in fruits such as oranges and tomatoes, as well as in potato tubers [70,71]. The significant variations (up to 1000-fold) in endogenous DOPA levels among plant species (Table 2) suggest that some DOPA functions may differ among plant organs and species.

### 3.3. Primary Function and Stress Responses

**Figure 3:**
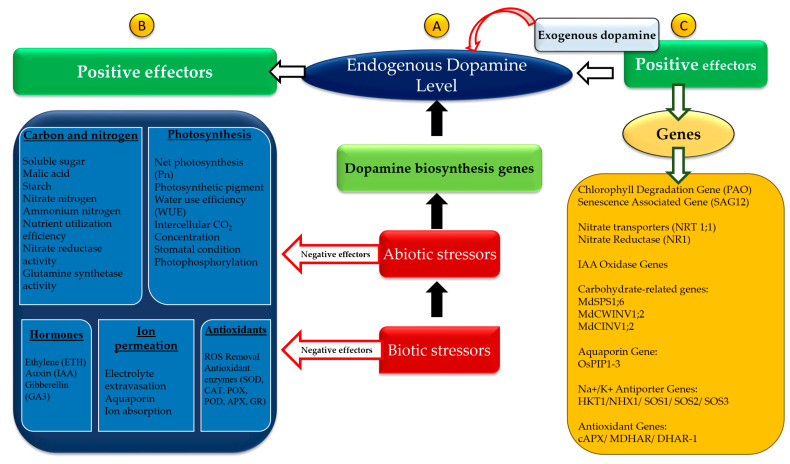
The role of dopamine in plant stress responses (Adapted from Liu et al. [72]).

The expression of genes involved in dopamine biosynthesis in plants can be induced by drought, salinity, and disease, leading to elevated levels of endogenous dopamine (Figure 3A). Dopamine plays a critical role in plant growth, development, and stress mitigation, particularly in response to various abiotic stresses [72]. It modulates the expression of stress-related genes, including those associated with chlorophyll degradation, senescence, nitrate transport, IAA oxidase, aquaporins, and carbohydrate metabolism [1] (Figure 3C). Both endogenous and exogenously applied dopamine have been shown to reduce plant damage from a variety of abiotic and biotic stresses (Figure 3B). Nonetheless, several areas require further investigation. The specific genes involved in dopamine biosynthesis and metabolic pathways in plants are not yet fully characterized. While research has largely focused on exogenous dopamine application, the effects of increasing endogenous dopamine levels through transgenic approaches remain underexplored. Additionally, the study of catecholamine receptors in plants may provide insights into their molecular mechanisms. Although dopamine receptors have not been identified in plants, experiments suggest the presence of catecholamine receptors. Marchiosi et al. [73] identified a class of DoH-CB proteins that regulate catecholamine activity and can bind to dopamine, an interaction facilitated by auxin induction. It is proposed that DoH-CB proteins may act as catecholamine receptors in plants, with auxin enhancing their binding affinity [74]. In summary, while research into dopamine’s role in enhancing plant stress tolerance is emerging, it holds promise for developing new stress-resistant plant varieties.

DOPA plays a significant role in the regulatory frameworks of ion homeostasis, oxidative radicle detoxification, and hormonal metabolic activities. In soybean, DOPA increased the SOD interaction in soybean roots, resulting in reduced ROS production and lipid peroxidation [75]. Similarly, DOPA decreased H_2_O_2_ production in leaves under drought stress [1]. In addition, DOPA regulates the production of phenylpropanoids as well as the activity of phenylalanine ammonia lyase (PAL) and tyrosine ammonia-lyase (TAL) in roots [76]. In particular, DOPA-induced root reduction has been linked to elevated auxin accumulation triggered by the suppression of IAA oxidase [77]. In *Papaver somniferum*, it has been reported that dopamine levels in latex and cell-free extracts differ with organ development stages [43].

Microarray data revealed that DOPA regulates the expression of more than 170 genes associated with abiotic and biotic stresses [73]. In rice treated with 0.15 M NaCl, exogenous DOPA downregulated *OsPIP-1*, indicating its role in plasmalemma aquaporins and the amelioration of salt stress [78]. In Chinese crab apple (*Malus huphehensis*), application of 100 μM DOPA reduced stress symptoms in nutrient-deficient plants [65]. Correspondingly, in the same species, exogenous DOPA enhanced nutrient uptake under drought stress and suppressed genes associated with senescence [79]. DOPA serves as a water-soluble antioxidant that induces cellular oxidative relief, which is characterized by an increase in the transcription of antioxidative enzymes in the ascorbate–glutathione cycle. It has been shown that exogenous DOPA greatly decreases H_2_O_2_ buildup (~30%), Malondialdehyde (MDA) content (15%), and Electrolyte Leakage (EL) accumulation (20%); it also enhances the activities of SOD (50%), CAT (~2-fold), and POD (2.2-fold) enzymes, compared to untreated control plants [35]. Similarly, apple trees treated with 100 μM exogenous DOPA showed enhanced resistance to drought stress, as well as nutrient uptake and transport, and plant growth [79]. Likewise, DOPA supplementation improved chlorophyll levels, stomatal function, and photosynthesis in apple trees. Furthermore, DOPA treatment enhanced the anti-senescence response in rice plants by regulating the expression of genes involved in nutrient uptake, transport, and restoration [80]. Another study on watermelon reported that application of 100 μM DOPA improved seedling health by reducing chilling stress through regulation of polyamine (PA) metabolism and proline content, as well as POD, SOD, and CAT activities [81].

To further improve plant resilience and stress tolerance, DOPA also controls the expression of genes related to DOPA synthesis and breakdown, leaf aging, sugar metabolism, salt stress response, nitrate uptake, water channel (OsPIP), auxin (IAA oxidase), and the antioxidant enzymes cAPX, cGR, Monodehydroascorbate Reductase (MDHAR), and Dehydroascorbate Reductase (DHAR-1) [82]. Therefore, DOPA plays significant roles in a wide range of plant stress responses, physiological processes, and overall growth and development.

Because of its pro-oxidative properties, DOPA promotes the formation of growth inhibitors such as melanin, free radicals (including semiquinone), and ROS bursts [83]. In roots, DOPA-induced lignin formation is associated with decreased H_2_O_2_ levels [82]. Therefore, DOPA influences root elongation and regulatory oversight via diverse pathways. An investigation of differentially expressed genes (DEGs) on *Malus domestica* pretreated with 100 μM DOPA revealed that DOPA regulates the expression of genes associated with nitrogen, amino acids, and secondary compounds during water deficiency stress [84]. This study also showed that DOPA serves as a main regulator of several key transcription factors including NAC, ERF, and WRKY.

## 4. Gamma-Aminobutyric Acid

### 4.1. Synthesis and Degradation

**Figure 4:**
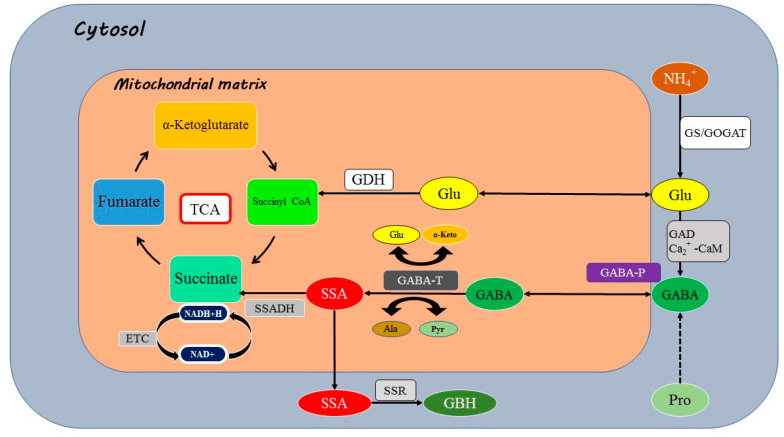
The role of the gamma-aminobutyric acid (GABA) shunts in plant metabolism: synthesis of GABA from glutamate and its mitochondrial metabolism. Ca^2+^/Calmodulin complex (Ca^2+^/CaM), glutamate decarboxylase (GAD), GABA permease (GABA-P), GABA transaminase (GABA-T), succinic semialdehyde dehydrogenase (SSADH), succinic semialdehyde reductase (SSR), pyruvate (Pyr), α-ketoglutarate (αKG), succinic semialdehyde (SSA), c-hydroxybutyric acid (GBH), electron transport chain (ETC). (Adapted from Ramos-Ruiz et al. [92]).

GABA is a non-protein amino acid that is used by plants as a signaling molecule [85]. This molecule is synthesized in the plant cell cytosol from glutamic acid by the enzyme glutamate decarboxylase (GAD) (Figure 4), the slowest step in its production process [86]. GABA transaminase (GABA-T) utilizes glyoxylate and pyruvate as acceptors for amino groups, resulting in the formation of glycine and alanine [87]. In tomato fruit, GABA-T prefers 2-oxoglutarate over pyruvate, resulting in glutamate recovery for the GAD response. As a result, succinate produced by succinate semialdehyde dehydrogenase (SSADH) can either join the TCA cycle or act as an electron donor in the mitochondrial electron transport chain [88]. Except for SSADH, several isoforms of the enzymes involved in GABA biosynthesis have been identified in different plant species [89]. In plants, GAD possesses a calmodulin (CaM) binding domain, and GAD activity is activated by the Ca^2+^/CaM complex at physiological pH (7.0–7.5) in vitro. In contrast, at acidic pH, GAD activity remains largely unaffected by Ca^2+^/CaM, demonstrating an optimal pH at 5.8 [86]. After synthesis, GABA is transported into vacuoles or to the extracellular space, where it can interact with its receptors on the tonoplast or plasma membrane [90]. GABA is degraded by GABA-T to succinic semialdehyde, which in turn, is metabolized to succinate and glutamate [91].

### 4.2. Content in Plant Tissues

GABA-containing cells have been identified in different plant tissues, including flowers, leaves, shoots, and roots. The GABA released into extracellular spaces can interact with the G protein-coupled receptors in other cells [93], activating a signaling cascade that can lead to a number of cellular responses [94]. Studies conducted over the last three decades revealed a range of GABA concentrations in plants ranging from micromolar to millimolar levels [95], and this varies across plant tissues and developmental stages [95,96]. In general, GABA concentrations are higher in young, actively growing tissues compared to mature or aged tissues. This is likely because developing tissues such as root tips and young leaves, which are responsible for nutrient uptake and photosynthesis, respectively, have greater metabolic rates, and therefore, require increased GABA production [97]. It has also been shown that GABA is differentially distributed within plant tissues. In roots, GABA is primarily present in the root cap and elongation zone, where it controls root architecture and development. In leaves, GABA is concentrated in the mesophyll cells, where it contributes to photosynthesis and stomatal control. Also, GABA levels rise in the pistil during pollen germination and fertilization, directing pollen tube growth and mediating interactions between pollen and the ovule [98].

Biotic and abiotic stresses such as herbivory, salinity, and drought cause a rise in GABA levels. These stress responses are mediated by various mechanisms, including the activation of GABA biosynthetic enzymes and the inhibition of GABA degradation pathways [99]. Furthermore, the accessibility of essential nutrients, in particular nitrogen and sulfur, can influence GABA synthesis and metabolism. Deficiencies in these nutrients can lead to reduced GABA levels [37].

### 4.3. Primary Function and Stress Responses

GABA is a crucial signaling molecule in plants, contributing directly to regulation of the activity of plant-specific anion transporters, thereby, modulating plant growth and development [96]. This function is further supported by evidence that exogenous GABA, beyond its role as a nutrient, has specific effects on gene expression and physiological responses in plants [100]. The identification of GABA as a plant signaling molecule has provided lines of inquiry into plant development, stress tolerance, and crop improvement. Furthermore, it has been proposed that GABA may influence interactions between plants and various organisms like fungi, bacteria, and insects, affecting plant growth regulation and defense mechanisms [100]. The identification of a crop glutamate receptor (GLR) family provoked a hypothesis that GABA may function as a signal through GLRs [101]; however, no GABA-interacting GLR has been identified so far. Stressors such as salt, cold, heat, or drought cause calcium ions to flow into plant cells through calcium channels, raising their concentration in the cytosol. This triggers ROS production, which in turn causes a further increase in cytosolic calcium levels. Ultimately, this cascade of events leads to GAD activation by calcium through its interaction with the CaM binding site (CaMBD), directing the conversion of glutamate to GABA. It has been shown that the key enzymes involved in the tricarboxylic acid cycle (TCA) are suppressed under deficit [102]. Therefore, GABA plays an essential role in ion transport regulation across the plasma membrane and tonoplast. This regulation is crucial for preserving the osmotic balance and promoting cell signaling processes [97]. Furthermore, GABA has been shown to increase stress tolerance in plants by regulating the expression of genes associated with ROS production, signal transduction, and stress–response pathways [103].

Recent studies have demonstrated that GABA plays a significant role in the regulation of the aluminium-activated malate transporter (ALMT) protein family activity, impacting plant responses to drought and acidic/alkaline soils [104]. Consequently, GABA accumulation under stress conditions may contribute to the modulation of plant stress tolerance mechanisms by affecting the expression and function of essential enzymes and ion channels. An important step toward establishing experimental evidence for the function of GABA as a signaling molecule in plants has been the identification of a putative mammalian-like GABA binding site with 12 amino acids on the ALMT class of plant anion channels [104]. This region was identified as a GABA receptor site in plants [105]. Principally, ALMT proteins have been documented to encode voltage-gated anion channels, with the additional inclusion of fast-acting anion channels [106]. Members of the ALMT protein family have been involved in vacuolar homeostasis (AtALMT9), aluminium tolerance (TaALMT1), stomatal movement (AtALMT12), mineral nutrition and ion homeostasis (ZmALMT1), and soil nutrient solubilization (AtALMT12 and ZmALMT2) [107]. Cell wall depolarization and the generation of ionic species (such as malate) are brought about by the activation of the ALMT ionic connection [108]. Additionally, anions like sulfate have the ability to transactivate the ALMTs on the efflux side of the protein channel [96]. Even though both GABAA effectors and ALMTs encode for ionic pathways, their corresponding coding regions differ greatly. ALMTs have a 12-amino-acid extension near the C terminus just at the end of the sixth transmembrane region, which is believed to be a GABA binding site [95,96]. Additionally, ALMT-mediated GABA-regulated anion (malate) efflux is susceptible to low micromolar GABA concentration [96]. In wheat, for instance, ALMT1 activation in planta heterologous expression systems resulted in a negative relationship between ionic outflow (malate) and intracellular GABA levels. Alterations in the aromatic amino acids tyrosine (Y) and phenylalanine (F) in the putative cysteine binding site of GABA change its appreciation and raise the EC50 from 3.4 μM to 1.8 mM in wheat ALMT1 and from 6.0 M to 380 M in a *Vitis vinifera* ALMT9 homolog [95].

It has been shown that GABA application stimulates the glyoxalase system and antioxidant enzymes, which are critical for the detoxification of methylglyoxal [109]. Exogenous GABA supplementation can boost plant growth by stimulating the endogenous levels of GABA, hormones, and amino acids through the regulation of important genes in plant hormone biosynthetic pathways [107]. GABA is also essential for controlling the transcription of genes involved in the antioxidant system during plant development, which helps to reduce oxidative damage [82]. Moreover, GABA treatment affects TCA enzymes, influencing respiratory metabolism [110]. A recent study showed that chlorophyll production is altered by the application of exogenous GABA [111]. In muskmelon, in addition to assisting seedlings in maintaining their osmotic balance, GABA has been shown to have an impact on the ascorbic acid–glutathione cycle and the accumulation of hydrogen peroxide, making plants more tolerant to saline–alkali stress [112]. Another study using *Caragana intermedia* showed that under saline conditions, exogenous GABA can reduce the production of hydrogen peroxide by regulating the expression of genes encoding hydrogen peroxide-producing enzymes [113]. Table 3 gives details of plants in which GABA has been utilized to improve abiotic stress tolerance.

Abiotic stress stimulates GABA accumulation through two distinct mechanisms. Firstly, acidification of the cytosol disrupts physiological and structural processes, resulting in the activation of GAD and the formation of GABA [132]. GABA accumulation was enhanced in hypoxic soil due to a significant drop in cytosolic pH [132]. Secondly, GABA production is sensitive to changes in calcium concentrations triggered in response to abiotic stress factors such as excess temperature, salt, and drought. Under these conditions, cytosolic Ca^2+^ levels rise quickly, inducing GABA production through CaM-dependent activation of GAD [133]. As a result of the advantageous role of GABA shunting, exogenous GABA application triggers a prolonged increase in endogenous GABA levels [134]. Studies on field crops such as wheat, rice, lentils, and melon show that exogenous GABA effectively delayed plant growth arrest induced by harmful environmental stresses, including high temperature [135], light or oxygen [92], salt [136], and drought [137]. Applications of external GABA improved photosynthetic capacity and anti-oxidative enzymes in stressed crops, resulting in decreased MDA and ROS levels, indicators of oxidative stress [138,139]. Lastly, the membrane integrity of plants treated with GABA is maintained under stress conditions [140,141].

## 5. Melatonin

Both animals and plants contain the pleiotropic indole molecule known as melatonin (*N*-acetyl-5-methoxy tryptamine) [113,142]. In plants, melatonin acts as a putative hormone that regulates plant development and growth under biotic and abiotic stress conditions [143,144,145,146,147,148]. Curiously, melatonin and its bioactive derivatives function by exchanging reactive nitrogen (RNS) and oxygen species (ROS) [149]. Melatonin seems to rapidly pass through the cell wall and be absorbed by the nucleus and mitochondria [150]. This intense non-receptor-mediated compound protects tissues and cells from oxidative stress by improving the antioxidant capacity of the organism and scavenging ROS and/or free radicals [150]. It, therefore, serves as the first defense mechanism against potential damage [151]. Melatonin has been found in algae, fungi and a broad range of angiosperms, including monocots and dicots [152]. Melatonin is widely distributed within plant organs, especially roots, flower petals, fruits, seeds, and bulbs; and examples of melatonin-containing plants are cucumber, tomato, apple, banana, onion, and rice [153].

Melatonin was first discovered in 1958 in the bovine pineal glands of cows. It is now acknowledged as a well-studied organic compound in a variety of biological systems ranging from microorganisms to mammalian species [144,154]. Being an eco-friendly chemical, melatonin may be a cost-effective tool for inducing biotic stress protection in plants. Studies on animals have revealed that melatonin possesses several therapeutic properties such as antioxidant, anti-inflammatory, immunomodulatory, and neuroprotective roles, making it an interesting option for the treatment of microbial infections. Correspondingly, recent studies have confirmed the beneficial role of melatonin in plant–pathogen interactions. The melatonin effects on epidermal and endosperm cells of onion [155] and bulbous plants [156] have opened up new research directions.

### 5.1. Biosynthesis and Degradation

**Figure 5:**
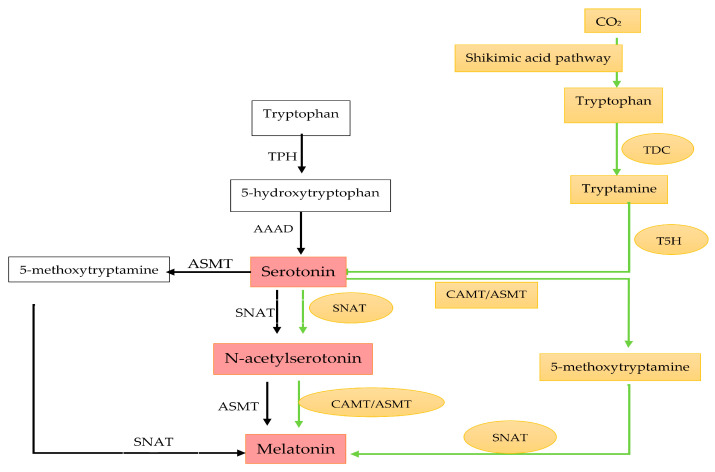
Comparative analysis of melatonin biosynthetic pathways in animals and plants highlights distinct enzymatic processes. TPH: tryptophan hydroxylase; AAAD: aromatic amino acid decarboxylase; SNAT: serotonin N-acetyltransferase; ASMT: N-acetylserotonin O-methyltransferase; TDC: tryptophan decarboxylase; T5H: tryptamine 5-hydroxylase; CAMT: caffeic acid O-methyltransferase. Color Coding (Black: enzymes that are present only in animals, Orange: enzymes that are present only in plants, Red: enzymes that are found in both animals and plants (Adapted from Tan et al. [159]).

The conversion of serotonin to melatonin involves a two-step process requiring three enzymes: serotonin N-acetyltransferase (SNATs), acetylserotonin O-methyltransferases (ASMTs), and caffeic acid O-methyltransferase (COMT). Among these, SNAT—formerly known as arylalkylamine N-acetyltransferase (AANAT)—is the most extensively studied and is widely recognized as the rate-limiting enzyme in the melatonin biosynthetic pathway. SNAT’s activity is critical for regulating melatonin production, while ASMT and COMT function as methyltransferases [157]. The sequence in which these enzymes act may vary, as all three demonstrate substrate affinity for 5-methoxytryptamine, N-acetylserotonin, and serotonin (Figure 5) [158].

Melatonin can be converted into other metabolites, including 5-methoxy tryptamine (5-MT), cyclic 3-hydroxy melatonin (3-OHM), N1-acetyl-5-methoxykynuramine (AMK), and N1-acetyl-N2-formyl-5-methoxykynuramine (AFMK) [160,161]. It is converted to AFMK by several enzymes, including eosinophil peroxidase (EPO), indoleamine 2,3-dioxygenase (IDO), myeloperoxidase (MPO), horseradish peroxidase (HRP), NADH-quinone oxidoreductase (NQR2), and cytochrome P450 (CYP) sub-forms. In addition to the subsequent CYPs’ activity on melatonin and its conversion into NAS (N-acetylserotonin), OHM (6-hydroxymelatonin), or AFMK, there is evidence of some additional pathways involved in the structural modification of AFMK and AMK [160]. These biologically active metabolites of melatonin enhance its mechanisms of action within plants [149].

Current studies on the molecular regulation of melatonin have largely focused on two model plants, rice and *Arabidopsis*, and there are only a few studies for other plants. The intricate mechanisms involved in the melatonin metabolic pathways are still poorly understood. However, these pathways are likely to include the degradation of enzymes, pseudoenzymes, and free radicals, and under extreme stress pressure, the primary pathway for melatonin degradation may involve free radical-based interaction [162]. Melatonin has been demonstrated to control its own production and breakdown, which improves plant development by regulating the uptake of mineral nutrients and stimulating certain mineral nutrition signal transduction [163,164]. Typically, melatonin degradation into several metabolites occurs through enzymatic and non-enzymatic pathways [165,166]. In the enzymatic pathway in animals, melatonin is first metabolized into 6-hydroxymelatonin (6-OHM) by the cytochrome P450 enzyme. Then, 6-OHM is sulfated by several enzymes to build 6-sulfatoxymelatonin (aMT6s) and AFMK [167,168]. It has been demonstrated that there are certain similarities between enzymatic melatonin degradation in plants and other eukaryotes [169]. The melatonin 2-hydroxylase (M2H) pathway appears to be the main catabolic pathway, playing significant roles in regulating the biochemical and physiological processes of plants under abiotic and biotic stresses [169]. Notably, the M2H and M3H enzymes are exclusive to land plants, as evidenced by their endemic nature [170]. Typically, the most common hydroxylated forms of melatonin found in plants are 2-OHM and c3-OHM, whereas 6-OHM primarily occurs in mammals. Additionally, in rice seedlings, 5-methoxytryptamine is another bioactive metabolite that is produced from serotonin by ASMT and from melatonin by N-acetylserotonin deacetylase (ASDAC) [165].

In the non-enzymatic route, different oxidant free radicals and ROS degrade melatonin, resulting in the production of several metabolites, including AMK, AFMK, 2-OHM, 4-OHM, and cyclic 3-OHM (c3-OHM) [165,167]. Melatonin can be nitrosated, leading to the production of N-nitrosomelatonin [171,172]. The mitogen-activated protein kinase (MAPK) cascade pathway and other more complex signaling networks (hormones, ROS, and Ca^2+^) [165,173,174,175,176] are linked to numerous melatonin-mediated abiotic stress responses (e.g., drought, salt, and heat) and physiological processes (e.g., flowering, senescence, somatic embryogenesis, sugar metabolism, and secondary metabolism). By restoring the redox state, these metabolites may mitigate oxidative damage [177,178]. Nevertheless, little is known about the signaling routes that melatonin metabolites adopt in plants.

### 5.2. Content in Plants

Melatonin accumulation in plants is affected by genetic background, growth stage, and environmental factors [145]. The melatonin concentrations can vary considerably between plants, organs, and even their developmental stage [179], with concentrations ranging from nanograms to grams of fresh weight [180]. In a study on horticultural plants, melatonin levels differed significantly between species, with yarrow (*Achillea millefolium*) having the highest levels (340,000 pg/g) [181] and currant tomato (*Solanum pimpinellifolium*) having the lowest concentration (0.1 pg/g) [182]. Melatonin levels vary substantially amongst different varieties of the same species, as observed for eight grape cultivars ranging from 5 to 965 pg/g FW [183]. Within the same tomato variety, the lowest concentration of melatonin was in leaf/stems (2000 pg/g FW), and the highest (39,400 pg/g FW) in seeds [184]. In general, seeds contain the highest melatonin levels in plants, followed respectively by leaves, roots, flowers, and fruits [185]. Melatonin levels were observed to exhibit a young-to-mature-to-old senescence pattern in leaf tissues at different stages of growth [186,187]. Furthermore, transgenic regulation, special bacterial strain induction, exogenous melatonin administration, and stress conditions can affect endogenous melatonin levels [188,189,190].

### 5.3. Primary Function and Stress Responses

#### 5.3.1. Cell Signaling

Melatonin and the plant auxin indole acetic acid (IAA) are both indoleamines, sharing the same precursor, tryptophan. Consequently, it is assumed that melatonin must play a significant role in the regulation of plant development and growth. Previous studies have discovered that melatonin restricts the circadian rhythms of *Chenopodium rubrum* [191]. Moreover, the application of exogenous melatonin to this plant influenced flower development early in the light/dark cycle [4]. Following melatonin treatment, soybean plants exhibited significant increases in leaf size, plant height, pod size, and seed production, suggesting that melatonin may boost soybean growth and seed yield [192]. Melatonin has been shown to protect plants by reducing chlorophyll breakdown in barley leaf tissue [193]. Similar to IAA, melatonin has been demonstrated to promote the development of etiolated cotyledons in *Lupinus albus* [194].

**Figure 6:**
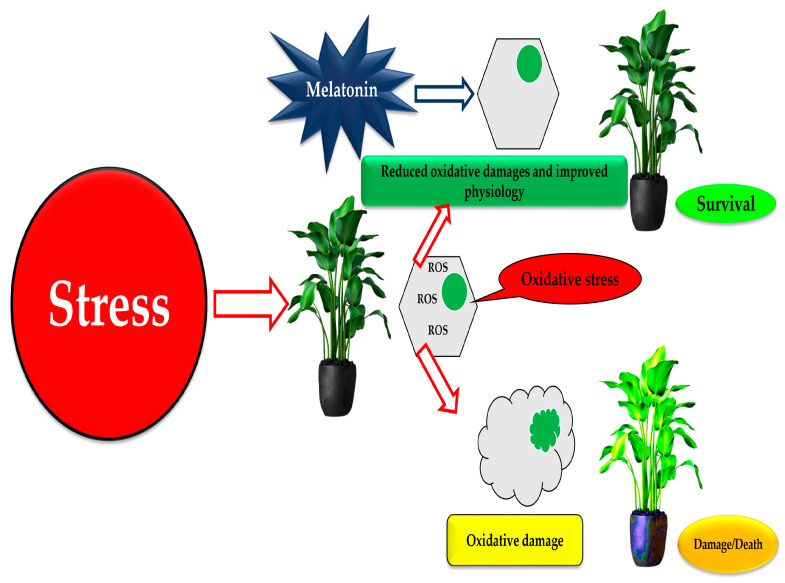
The role of melatonin in plant stress responses (Adapted from Colombage et al. [197]).

Melatonin neutralizes the highly toxic OH^•^, resulting in the production of effective antioxidant cyclic 3-OHM. This leads to better scavenging of superoxide (O_2_) and NO^•^ radicals and enhances the functions of many antioxidative enzymes, including both Mn- and Cu-superoxide dismutases (MnSOD and CuSOD), glutathione peroxidase (GPx), catalase, and glutathione reductase (GR). Although inhibited by c-glutamylcysteine synthase, melatonin-stimulated GSH synthase increases the concentration of intracellular GSH. Melatonin enhances plant tolerance to oxidative stress through two primary mechanisms: directly, by scavenging ROS and activating both enzymatic and non-enzymatic antioxidant systems [195]; and indirectly, by promoting physiological functions such as root and shoot growth and facilitating seed germination, often in synergy with plant growth regulators and photosynthetic processes. Melatonin also reduces membrane lipid peroxidation during oxidative stress, lowering MDA levels and minimizing electron leakage in plant cells, which collectively protects against programmed cell death [74]. The cumulative effect of the protective metabolic changes induced by elevated melatonin levels mitigates damage caused by abiotic stress in plants (Figure 6). Changes in gene expression have been observed in melatonin-responsive crops, including genes involved in osmotic adjustment, carbohydrate synthesis, tri-carboxylic acid transition and transport, hormone metabolic activity, metal homeostasis, and redox procedures [196,197].

Thus far, several studies have demonstrated that endogenous melatonin concentrations increase in stressed plants, and melatonin is crucial for enhancing plant tolerance to both biotic and abiotic stressors [197,198]. In *Arabidopsis*, melatonin treatment led to the expression of specific stress-related genes, including those involved in preserving chlorophyll content and regulating chlorophyll degradation via the light-regulated enzyme chlorophyllase (CLH1) [199]. Through the regulation of antioxidant enzymes, melatonin modulates stress-related gene expression, enhances photosynthetic activity, reduces modifications in leaf structure and function, and postpones metabolic synthesis and senescence, providing a well-known mechanism for plant protection under extreme conditions [198]. Melatonin is widely recognized for its protective role in plants under abiotic stress conditions, where it alleviates oxidative damage through antioxidant activity, regulates phytohormone levels, activates stress-responsive genes, and induces the expression of heat shock proteins and transcription factors [196,197,200]. Numerous studies have demonstrated that pre-treatment with exogenous melatonin significantly mitigates plant damage from various stresses, including drought [201,202,203,204], cold [205,206,207], heat [208,209,210,211,212,213,214,215,216], heavy metals [217,218,219], salt [220,221,222], and waterlogging [223,224,225]. Nonetheless, many aspects of melatonin in plants remain ambiguous, such as its metabolism and regulatory mechanism during stressful situations [226]. Research is still needed on the processes underlying the distinct distribution of melatonin in shoots and roots, as well as the roles of melatonin in plant organs.

#### 5.3.2. Regulation of Other Physiological Mechanisms

Crops respond to unfavorable environmental circumstance based on their physiological states. Melatonin, as an effective antioxidant and a powerful free radical scavenger [227], can modulate physiological activities in response to adverse environmental conditions. Melatonin is an amphiphilic or amphipathic molecule that easily penetrates through the cell membrane and accumulates in the cytosol, nucleus, and mitochondria [198,227]. Cyclic 3-OHM possesses antioxidant properties which can neutralize the toxic OH^•^ as well as scavenge superoxide residues (O_2_) [228]. Melatonin generates antioxidative products by interacting with hydrogen peroxide [229]. In vivo, such antioxidative activities cause a supportive effect [230], leading to the activation of several enzymatic antioxidants and an improvement in anti-oxidant effectiveness [231,232,233,234]. Melatonin acts as a direct antioxidant, reducing oxygen radical levels as compared to AsA. As mentioned previously, several melatonin metabolites, including AMK, AFMK, 2-OHM, and 3-OHM, serve as effective antioxidants in plants [151]. Melatonin mediates a variety of antioxidant pathways, including the SOD, glutathione ascorbate cycle, peroxidases, and catalase, to modulate plant responses to biotic and abiotic stresses [235]. For instance, melatonin can detoxify toxic reactive nitrogen species such as nitric oxide [231,232,233,234,236,237] as well as compensate ROS and RNS in cells [238]. The interaction of melatonin and ROS generates signaling molecules within plants, intensifying ROS responses [239]. In transgenic rice plants, the melatonin biosynthetic pathway alters phytohormone activity under harsh environments to promote root development [240]. In rice plants treated with abscisic acid and methyl jasmonate, the melatonin pathway is known to be involved in the articulation of ASMT-mRNA to mediate stress responses [236]. In addition, melatonin has been reported to promote the activity of plant antioxidant enzymes, including SOD, POD, CAT, and APX, to combat oxidative damage under drought stress [147,241]. Increased antioxidant enzyme activities mediate reductions in MDA and ROS, and hence melatonin is regarded as a broad-spectrum antioxidant and oxygen radical scrapper [229]. The involvement of antioxidant enzymes in controlling ROS in response to abiotic stressors has also been reported [147]. Studies show that foliar and/or soil application of melatonin on different plants improved their resistance to stress by protecting the photosynthetic machinery, boosting antioxidant potential, and enhancing water-holding capacity [147,192].

Furthermore, melatonin has been demonstrated to trigger somatic embryogenesis in coffee in in vitro cultures [17]. Melatonin has also been shown to affect leaf and branching structures in rice and tomato [242,243]. Also, exogenous melatonin has been shown to enhance plant biomass [192,240,244]. These studies suggest that besides its role in signaling and other plant growth and development regulatory cascades, melatonin plays a role in nutrient cycling.

## 6. Serotonin

Millions of years ago, serotonin—an ancient indoleamine molecule—likely had a role in maintaining the primary prokaryotic life systems as a potent antioxidant to counter the progressively oxygen-rich atmosphere [238]. It was first revealed to be a neurotransmitter signaling molecule in mammals [245], and many years later, it was identified in plants [246]. It is now been found in almost all plant families, where it is involved in many aspects of plant development and growth, such as regulation of reproductive development, energy acquisition, the control of root and shoot organogenesis, delaying senescence, and plant responses to abiotic and biotic stresses [247].

### 6.1. Biosynthesis and Degradation

**Figure 7:**
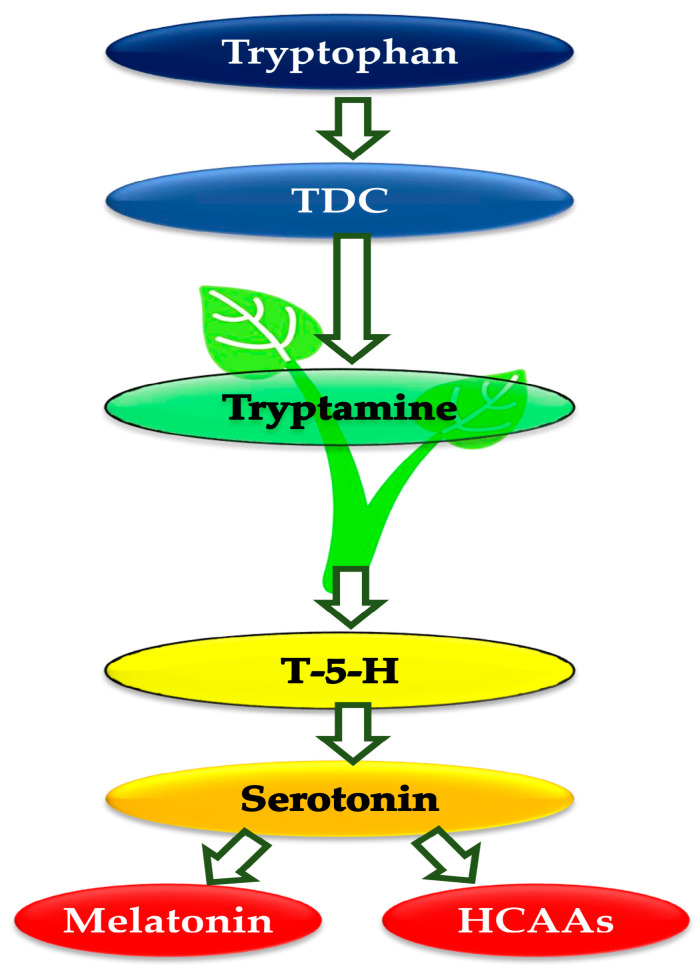
Biosynthetic pathway for serotonin and its key transformation products in plants. HCAA, hydroxycinnamic acid amide; T-5-H, tryptamine-5-hydroxylase; TDC, tryptophan decarboxylase (Adapted from Erland and Saxena [251]).

Plants initially produce tryptophan through the chorismate pathway. This tryptophan is then decarboxylated by tryptophan decarboxylase (TDC) to generate tryptamine, which is another bioactive amino acid and a common precursor for secondary metabolites [248]. Tryptamine is subsequently hydroxylated by tryptamine-5-hydroxylase (T-5-H) to produce serotonin (Figure 7) [249]. In plants, TDC is the rate-limiting enzyme that regulates the flow through the biosynthetic pathway. Experiments manipulating TDC in transgenic lines have revealed downstream changes in serotonin and melatonin levels. This could serve as a mechanism for directing tryptophan toward other crucial biosynthetic pathways, such as those for indole alkaloids, auxin, or protein synthesis [250]. While the biosynthesis and regulation of serotonin in vertebrates is well understood, research on serotonin biosynthesis and regulation in plants is still an emerging field and remains an area of active and promising investigation [247].

TDC and T5H are essential for serotonin synthesis, with TDC acting as a rate-limiting step due to its high Km with tryptophan (690 M) and imperceptible overexpression in untreated controls. In rice, TDC is nearly impossible to detect in leaf tissue, while, T5H is constitutively expressed [252]. Nonetheless, T5H and downstream enzymes, such as serotonin N-hydroxycinnamoyl transferase (SHT), exhibit lower Km values on the equivalent surfaces, indicating that the biosynthetic pathway of secondary metabolites produced from serotonin is limited to cell membrane phases with elevated tryptophan levels. Serotonin has been found in several crops and is more plentiful in seeds and fruits than vegetative organs [253]. Although several biological roles have been suggested for serotonin in crops [254], the exact functions remain to be thoroughly investigated using molecular, biochemical, and genetic approaches.

Unlike the well-known serotonin biosynthesis routes, less is known about its catabolic mechanisms in plants. It can be degraded by oxidative pyrrole ring cleavage, dealkylation, hydroxylation, and deacetylation reactions, according to information from dinoflagellates. Breakdown products include AFMK, 5-methoxytryptophol (5-M), 5-methoxyindole-3-acetic acid (5-MIAA), and 5-methoxytryptamine (5-MT). Nevertheless, these reactions have not been detected in higher plants [255,256,257].

### 6.2. Content in Plants

The serotonin concentration varies with plant species, cultivar, developmental phase, and tissue type. Serotonin levels are moderate to high in plant species such as *Carya ovata*, *Griffonia simplicifolia*, *Juglans cinerea*, *Solanum lycopersicum*, *Urtica dioica*, *Vaccinium* spp., *Zea mays*, *Zingiber officinale* and others. The highest concentrations of serotonin are found in fruits, vegetables, and seeds; and they also differ amongst cultivars of the same species [258]. Aerial parts of plants contain higher serotonin levels than underground organs such as potato tubers. An analysis of a significant number of plant families revealed that high levels of serotonin occur in developing flowers and seeds, varying with the developmental stage [30,186,232,259,260,261,262,263]. In fact, like melatonin, seeds are particularly rich in serotonin, as reported for many species, including within the *Clusiaceae*, *Fabaceae*, *Poaceae*, *Rubiaceae*, and *Solanaceae* families [184,185,264,265,266,267,268], indicating that reproductive tissues contain significantly higher concentrations of serotonin in comparison to vegetative tissues. Park et al. [269] reported significantly different serotonin concentrations in Merlot grapes across developmental stages, suggesting that, besides protective functions, serotonin may serve as a regulator of plant reproduction. Additionally, a rise in the endogenous serotonin content was associated with improved de novo shoot regeneration [26], demonstrating its role in modulating plant morphogenesis in vitro.

### 6.3. Primary Function and Stress Responses

#### 6.3.1. Cell Signaling

**Figure 8:**
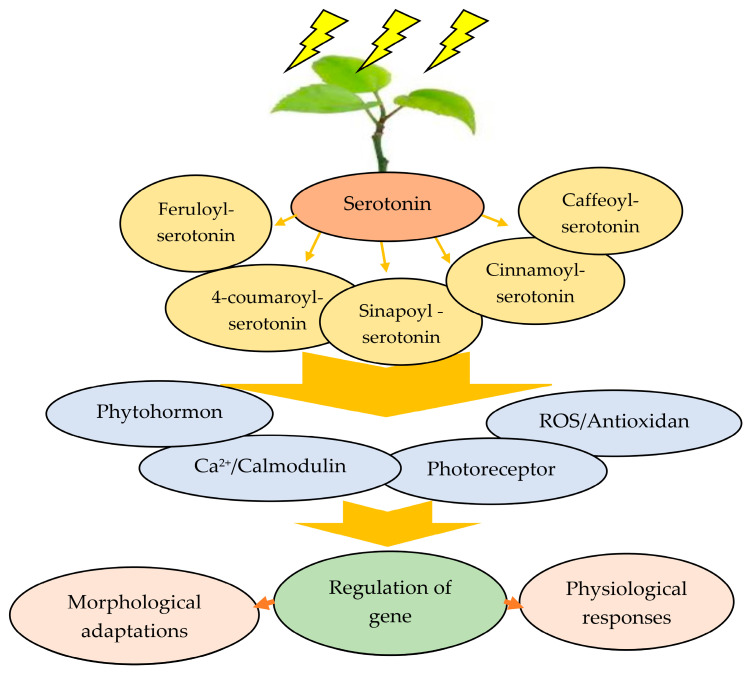
The roles of serotonin in plant responses to stress.

In addition to its involvement in melatonin biosynthesis, serotonin serves as a precursor for a number of metabolites that play crucial roles in plant protection under diverse stress conditions [270]. These products predominantly include feruloyl-serotonin, 4-coumaroyl-serotonin, caffeoyl-serotonin, cinnamoyl-serotonin, and sinapoyl-serotonin, which are classified as phenylpropanoid amides (PAs) (Figure 8) [270]. The synthesis of these compounds occurs through a condensation process between cinnamoyl-CoA thioesters and serotonin. Serotonin N-hydroxycinnamoyltransferase, located in the cytosol, is essential for the final stage of phenylpropanoid amide (PA) biosynthesis, which has been identified in over sixteen species belonging to eight plant families [67]. In coffee, serotonin is present in the vascular tissues of organs such as somatic mature roots, embryos, immature fruits, and stems [271]. Furthermore, investigations with the downstream enzyme SNAT in *Pyropia yezoensis* indicated that serotonin is limited or transported to chloroplasts before being converted to N-acetylserotonin and, later, melatonin [272].

Although serotonin receptors have not been found in plants, plants may have evolved to adapt their serotonergic systems to their environment. For instance, studies involving the use of known antagonists and agonists for serotonin receptors have been shown to modulate developmental processes in plants [27]. Much more research is required to understand the location of biosynthetic pathways and the mechanisms of serotonin actions in crop plants. These findings will assist scientists in discovering the possible role of serotonin during the early stages of crop domestication. Although research on the possible destination and synapses of melatonin in crops is limited, it suggests that another serotonin repressor may exist. So far, a specific crop protein, Hyp-1, a suspected pathogenesis-linked PR-10-type protein from *H. perforatum*, has been linked to melatonin [273]. Although serotonin was not investigated in this study, it could be a promising candidate for further research. Additionally, the COP1 and COP9 signaling molecules are thought to play crucial roles in mediating melatonin’s effects and may be downstream effectors of a hypothesized melatonin receptor [274]. Even though COP1 and COP9 were not found to be directly associated with serotonin signaling, their relationship to phytochrome and melatonin signaling—two processes that are known to interact with serotonin signaling in plants—indicates that they may be part of an additional serotonin signaling route. UVR8, a UV-B receptor, is another upstream inducer of COP9. Light-mediated effects on plant growth have been reported for serotonin [275]. It is possible that other light receptors and serotonin interact directly to facilitate phytochrome intervention [247]. With its repeating tryptophan residues as its chromophore, UVR8 is particularly relevant to serotonin. Tryptophan is an aromatic, naturally UV-active material that may absorb UV-B light and cause a receptor’s structure to alter, which in turn activates the COP9 signalosome. Serotonin has the same UV activity as tryptophan, pointing to the unproven but intriguing theory that serotonin functions as a photon detector in response to intense light and UV radiation.

#### 6.3.2. Regulation of Other Physiological Pathways

Serotonin is considered a non-growth regulator in plants with cytokinin-like activity that can stimulate lateral root growth in a dose-dependent manner, by promoting the formation of pre-existing lateral root buds [276]. It participates in several developmental processes, including root and shoot organogenesis and patterning, cell proliferation and differentiation, biomass yield, embryo development, and senescence. In *Hypericum perforatum*, shoot production has been linked to higher endogenous serotonin levels, and suppression of serotonin receptors resulted in a decrease in shoot biomass [277]. Serotonin has also been reported to promote shoot development in *Mimosa pudica*, with treatment greatly increasing shoot mass and length, root biomass, and overall fresh and dry weights of in vitro cultivated plantlets [145]. These effects were restored by inhibitors of serotonin receptors such as fluoxetine and p-chlorophenoxyacetic acid.

In coffee plants, calcium signaling has been found to play a key role in serotonin-mediated somatic embryogenesis [67]. When calcium inhibitors were applied, the growth-promoting effects of serotonin were markedly reduced, while calcium supplements restored plant growth. Thidiazuron treatment of *Echinacea purpurea* leaves increased endogenous serotonin levels, which led to an increase in callus production within the tissues [278]. The capacity of serotonin to enhance plant growth, increase coleoptile mass, and elongate hypocotyls, demonstrates its significance during embryonic growth and development. Although serotonin has been reported to promote root mass in walnut plantlets, in sunflower seedlings, however, it has been found to restrict hypocotyl and main root growth after two and four days of development [279], suggesting that serotonin may function in a dose-dependent manner.

In *Arabidopsis*, for instance, serotonin improved lateral root growth at low to moderate concentrations (10–160 µM), but impeded primary root growth, lateral root development, and hairy root formation at elevated levels (>160 µM), although adventitious root formation was improved [280]. Though serotonin stimulates root formation, it has also been reported in certain plant species to act as an auxin suppressor. In Arabidopsis, the application of 10–160 µM of serotonin resulted in cytokinin-like effects, disrupting auxin transport and inhibiting primary root growth [280]. The morphological responses are determined by the rebalancing of melatonin and serotonin, where reduced melatonin levels stimulate root and organ development, whereas higher amounts of serotonin improve shoot organogenesis in the presence of exogenous auxin [26].

Silencing of SNAT in rice seedlings has been shown to inhibit serotonin-to-melatonin conversion, leading to elevated serotonin content, and thereby faster coleoptile growth [252]. In *Caffea canephora* and *Mimosa pudica*, the addition of an inhibitor of serotonin to melatonin conversion resulted in elevated endogenous serotonin levels, leading to a reduction in somatic embryogenesis potential [17]. Similarly, shoot formation in in vitro cultures was reduced by the application of inhibitors of human serotonin transporters [26]. These findings demonstrate that serotonin’s cytokinin-like effects extend beyond their antagonistic interactions with auxin during root development, encompassing the stimulation of shoot formation and somatic embryogenesis in vitro. Correspondingly, the addition of serotonin showed a positive effect on somatic embryogenesis and shoot multiplication [17,27].

However, overexpression of TDC, which resulted in a 11–25-fold increase in serotonin levels (dependent on tissue type), did not show any major impact on phenotype [252]. Advantages in radicle development were also noticed in barley crops inoculated for 72 h with 10^−8^ M serotonin [276]. Serotonin enhances phosophoinositide (PI) turnover and mimics the red-light influence in maize by increasing *nitrate reductase* transcript stages and decreasing *phyI* transcript levels. Low light intensity was found to be more effective than light supplementation in restoring serotonin levels. The serotonin-to-melatonin ratio influences light-mediated effects on crops. Serotonin density was discovered to exhibit a distinct diurnal cycle, peaking throughout the day and dropping by more than 80% immediately after the onset of darkness [268]. In maize, serotonin has been identified as a phytochrome activator by acting as an alternative to far-red light during activation of the phytochrome signaling pathway [281]. This is most likely accomplished by raising calcium absorption and activating nitrate reductase, which changes the turnover of phosphatidylinositol downstream [282]. Consistently, serotonin has been shown to be a powerful mediator of phytochrome activity, a key element driving the plant circadian cycle.

## 7. Conclusions and Prospects

Over the last two decades, there has been substantial advancement in understanding the roles of NTs in higher plants. In particular, studies on melatonin- and serotonin-induced regulation of plant response to abiotic stress have provided valuable evidence of a variety of physiological mechanisms involved in plant development and defense. However, there are still significant gaps in our understanding of NTs in plants. Abiotic stress is assumed to induce melatonin-induced regulation of calcium-dependent signaling in crops. The exogenous application of melatonin to plants facing abiotic stress has resulted in important insights into the transcriptomic and genomic regulation of pathways associated with hormone levels, metabolic activities, and other signaling events. Nevertheless, alterations in the melatonin biosynthetic pathway and turnover in response to abiotic stress require further investigation. The identification of receptors involved in serotonin and melatonin uptake and transport in crops has the potential to improve our knowledge of stress response processes. The idea of enhancing crop stress tolerance by the use of exogenous GABA is important in practice. When GABA is utilized in stressed crops, it improves GABA shunt action, photosynthetic efficacy, endogenous GABA synthesis and anti-oxidative enzyme activity, while reducing MDA levels, an oxidative indicator of ROS and eventually, membrane integrity. Moreover, application of 10 mM GABA to the canopy of legume crops led to elevated levels of macronutrients, as well as Mn, Zn, and B, in comparison to the untreated crops. This suggests that GABA might be an effective tool that producers can use to biofortify grain legumes and cereals. There is considerable evidence that dopamine contributes to plant development and growth, as well as tolerance to abiotic stress. Water deficit, salinity, and certain pathogens can all raise the endogenous dopamine levels in plants, and exogenous dopamine administration has the potential to reduce the degree of damage brought on by biotic and abiotic stressors. However, the dopamine biosynthesis routes, receptors, and metabolic pathways in plants remain to be identified so that endogenous dopamine expression can be improved through molecular crop breeding.

## Figures and Tables

**Table 1:** The influence of melatonin and serotonin on plant growth and development.

Plant Species	Influence	Dose	Application Method	Reference
**Melatonin**				
*Hypericum perforatum*	Shoot propagation and root stimulation	50–200 µM	Spraying	[3]
*Chenopodium rubrum Arabidopsis thaliana*	Prevent flowering	100–500 µM	Spraying	[4]
*Lupinus albus*	Extend hypocotyl length	0.01–1000 µM	Incubation in solution	[5]
*Lupinus albus*	Lateral and adventitious root regeneration	0.1–10,000 µM	Applying into agar blocks placed on top of hypocotyl	[5]
*Phaseolus vulgaris*	Improve salinity stress tolerance	100–200 µM	Spraying	[6]
*Cucumis sativus*	Increase seed germination	1 µM	Seed treatment	[7]
*Vitis vinifera*	Promote seed germination and emergence performance	1–25 µM	Seed immersion	[8]
*Prunus cerasus*	Promote the formation of adventitious roots	1 µM	In vitro application	[9]
*Arabidopsis*	Reduce meristem size	600 µM	In vitro application	[10]
*Arabidopsis thaliana*	Increase lateral roots	200–500 µM	In vitro application	[11]
*Vigna radiata*	Increase root elongation	20 and 100 µM	Seed immersion	[12]
*Brassica juncea*	Increase root number	0.1 µM	Seedling treatment	[13]
*Oryza sativa*	Increase adventitious roots	20 µM	Seedling treatment	[14]
*Brassica juncea*	Inhibit primary root development	100 µM	Seedling treatment	[13]
*Helianthus annus*	Increase hypocotyl length	5–15 µM	Seedling treatment	[15]
*Triticum aestivum, Avena sativa, Hordeum vulgare*, *Phalaris canariensis*	Enhance coleoptile growth	0.01–100 µM	Coleoptile section treatment	[16]
*Coffea canephora*	Enhance somatic embryogenesis	100 µM	In vitro application	[17]
*Arabidopsis thaliana*	Delay leaf senescence	20–125 µM	Watering	[18]
*Malus domestica*	Delay leaf senescence	10 mM	Detached-leaf treatment	[19]
*Zea mays*	Prevent seedling development			[20]
*Solanum lycopersicum*	Promote lateral roots development, root hair growth, biomass accumulation, and root activity	10–30 µM	Seedling treatment	[21]
*Chara australi* *s*	Increase photosynthesis efficiency	10 µM	Addition to artificial pond water	[22]
*Glycine max*	Enhance seed germination and development	20–100 µM	Seed treatment	[23]
*Solanum lycopersicum*	Promote post-harvest fruit ripening and quality	50 µM	Fruit treatment	[24]
*Malus domestica*	Promote post-harvest fruit ripening	50 µM	Fruit treatment	[25]
*Chenopodium rubrum*	Modify flowering period	100 and 500 µM	Seedling treatment	[4]
**Serotonin**				
*Hypericum perforatum*	Increase shoot production	100–200 µM	In vitro application	[26]
*Mimosa pudica*	Enhance shoot induction and growth	100–200 µM	In vitro application	[27]
*Coffea canephora*	Enhance somatic embryogenesis	100 µM	In vitro application	[17]
*Oryza sativa*	Delay senescence	0.8 µM	Transgenic over-expression	[28]
*Helianthus annuus*	Increase root length	15 µM	Seedling treatment	[15]
*Arabidopsis thaliana*	Inhibit main root growth, improve growth of lateral roots, and formation of adventitious roots	10–160 µM	Seedling treatment	[29]
*Helianthus annuus*	Enhance hypocotyl elongation	5–15 µM	Seedling treatment	[15]
*Datura metel*	Protect reproductive tissues	-	Endogenous elevation	[30]
*Hordeum vulgare*	Increase root weight, coleoptile weight, and mitotic index	0.8 µM	Seed treatment	[31]
**Dopamine**				
*Malus hupehensis*	Mitigate nitrogen stress	100 µM	Watering	[32]
*Solanum lycopersicum*	Mitigate the effects of salinity stress, promote plant and root growth	100 µM	Seedling treatment	[33]
*Pyrus communis*	Increase disease resistance	100 µM	Leaf treatment	[34]
*Malus hupehensis*	Promote alkali tolerance of seedlings	100 µM	Seedling treatment	[35]
*Cucumis sativus*	Enhance antioxidant capacity and improve disease resistance	100 µM	Seedling treatment	[36]
**Gamma-aminobutyric acid**				
*Vigna radiata*	Improve salinity tolerance	1.5 mM	Seedling treatment	[37]
*Punica granatum*	Improve photosynthesis efficiency	10–40 mM	Foliar application	[38]
*Andrographis paniculata*	Alleviate nitrogen deficiency stress	5 mM	Plant treatment	[39]
*Zea mays*	Enhanced plant growth	1 mM	Seedling spraying	[40]
*Lavandula dentata*	Alleviate the adverse effects of salinity and improve vegetative growth and root characteristics	20 and 40 mM	Foliar application	[41]
*Solanum lycopersicum*	Enhanced salt stress resistance	5 mM	Watering	[42]

**Table 2:** Dopamine levels in plant parts (Liu et al. [72]).

Plant Species	Plant Part	Dopamine Concentration μg/g FW
*Musa acuminatum*	Fruit peel	100
*Musa acuminata × M. balbisiana*	Pulp	55
*Musa acuminata*	Pulp	42
*Lolium perenne*	Seeds	37.66
*Musa acuminatum*	Pulp	2.5–10
*Solanum tuberosum* var. Desiree	Leaves	2–7
*Malus domestica*	Root	5–6
*Plantago major*	Pulp	5.5
*Persea americana*	Pulp	4
*Theobroma cacao*	Powder	1
*Brassica olereacea* var. italica	-	1
*Brassica olereacea* var. gemmifera	-	1
*Citrus sinensis*	-	<1
*Lycopersicon esculentum*	-	<1
*Solanum melongena*	-	<1
*Spinacia oleracea*	Leaves	<1
*Solanum tuberosum* var. Desiree	Tubers	<0.5

**Table 3:** Examples illustrating enhanced abiotic stress tolerance in plants from the exogenous application of GABA.

Plant	Abiotic Stress	Effects That Have Been Reported	References
*Solanum lycopersicum* and *S. melongena*	Heavy metal	Simultaneous application of GABA and nitric oxide (NO) reduced the harmful effects of heavy metals.	[114]
*Malus pumila*	Cadmium	Exogenously applied GABA significantly decreased the flow rate of cadmium ions (Cd^2+^) into d roots, resulting in a lower cadmium concentration in roots.	[115]
*Malus domestica*	Drought	GABA treatment of plants under drought stress improved the quality of the fruit and changed the levels of GABA and polyamines inside plants.	[116]
*Malus hupehensis*	Drought	GABA at a concentration of 0.5 mM was most effective for relieving plants under drought stress. It decreased the accumulation of hydrogen peroxide and superoxide anions in leaves during drought stress, while the activities of the antioxidant enzymes were increased.	[117]
*Nigella sativa*	Drought	Exogenous GABA application enhanced the growth and productivity of black cumin under water deficit stress.	[118]
*Trifolium repens*	Drought	Increasing endogenous GABA levels by external GABA application may improve drought tolerance in white clover by favorably regulating the GABA-shunt pathway and the metabolism of proline (Pro) and polyamines (PAs).	[119]
*Vitis vinifera*	Water deficit	GABA aggregates in high concentrations in the tendrils and stimulates their coiling independently of jasmonates.	[120]
*Citrus sinensis*	Water deficit	Exogenous GABA boosted endogenous GABA levels, improved respiration, and upregulated phytohormone biosynthesis genes, demonstrating that GABA collaborates with phytohormones to decrease plant stress.	[121]
*Lactuca sativa*	Salinity	Under salt stress, GABA improved seed germination and plant development, photosynthesis efficiency, antioxidant enzyme activities, and regulated hydrogen peroxide levels.	[122]
*Fragaria × ananassa*	Salinity	Physiological and molecular responses to salinity were improved by lowering ROS levels, enhancing antioxidant enzyme activity, and over-expression of stress-responding genes.	[123]
*Trifolium repens*	Salinity	GABA alleviated the negative effects of salt on seed germination.	[124]
*Morus multicaulis*	Salinity	External application of GABA to transgenic mulberry plants resulted in a significant increase in the activity of antioxidant enzymes and a reduction in ROS-related damage under salinity stress.	[125]
*Agrostis stolonifera*	Heat	Exogenous GABA supplementation largely prevented heat damage, increased water content, stabilized cell membranes in leaves, and improved photosynthesis.	[126]
*Agrostis stolonifera*	Heat	Through enhanced antioxidant activity, GABA treatment significantly decreased heat stress-induced damage and chlorophyll loss.	[127]
*Camellia sinensis* L.	Heat	GABA plays a key role in polyphenol accumulation and the antioxidant system activation in tea plants during heat stress.	[128]
*Solanum lycopersicum*	Chilling	GABA treatment protected tomato seedlings from cold stress by protecting membrane integrity via enhanced antioxidant enzyme activity and reduced MDA levels.	[129]
*Musa* spp.	Chilling	Applying GABA to banana fruit enhanced the antioxidant defense system and proline formation, which both helped to mitigate the damage caused by cold.	[130]
*Prunus persica*	Chilling	The administration of GABA to cold-stressed peach plants decreased total soluble solids content and thereby reduced the chilling injury index (CI) as well as the weight loss rate.	[131]
